# COVID-19 Vaccine Safety in Children Aged 5–11 Years — United States, November 3–December 19, 2021

**DOI:** 10.15585/mmwr.mm705152a1

**Published:** 2021-12-31

**Authors:** Anne M. Hause, James Baggs, Paige Marquez, Tanya R. Myers, Julianne Gee, John R. Su, Bicheng Zhang, Deborah Thompson, Tom T. Shimabukuro, David K. Shay

**Affiliations:** ^1^ CDC COVID-19 Response Team; ^2^ Food and Drug Administration, Silver Spring, Maryland.

On October 29, 2021, the Food and Drug Administration (FDA) amended the Emergency Use Authorization (EUA) for Pfizer-BioNTech COVID-19 (BNT162b2) mRNA vaccine to expand its use to children aged 5–11 years, administered as 2 doses (10 *μ*g, 0.2mL each) 3 weeks apart (*1*). As of December 19, 2021, only the Pfizer-BioNTech COVID-19 vaccine is authorized for administration to children aged 5–17 years (*2*,*3*). In preauthorization clinical trials, Pfizer-BioNTech COVID-19 vaccine was administered to 3,109 children aged 5–11 years; most adverse events were mild to moderate, and no serious adverse events related to vaccination were reported (*4*). To further characterize safety of the vaccine in children aged 5–11 years, CDC reviewed adverse events after receipt of Pfizer-BioNTech COVID-19 vaccine reported to the Vaccine Adverse Event Reporting System (VAERS), a passive vaccine safety surveillance system co-managed by CDC and FDA, and adverse events and health impact assessments reported to v-safe, a voluntary smartphone-based safety surveillance system for adverse events after COVID-19 vaccination,* during November 3–December 19, 2021. Approximately 8.7 million doses of Pfizer-BioNTech COVID-19 vaccine were administered to children aged 5–11 years^†^ during this period; VAERS received 4,249 reports of adverse events after vaccination with Pfizer-BioNTech COVID-19 vaccine in this age group, 4,149 (97.6%) of which were not serious. Approximately 42,504 children aged 5–11 years were enrolled in v-safe after vaccination with Pfizer-BioNTech COVID-19 vaccine; after dose 2, a total of 17,180 (57.5%) local and 12,223 systemic (40.9%) reactions (including injection-site pain, fatigue, or headache) were reported. The preliminary safety findings are similar to those from preauthorization clinical trials (*4*,*5*). The Advisory Committee on Immunization Practices (ACIP) recommends the Pfizer-BioNTech COVID-19 vaccine for children aged 5–11 years for the prevention of COVID-19 (*6*). Parents and guardians of children aged 5–11 years vaccinated with Pfizer-BioNTech COVID-19 vaccine should be advised that local and systemic reactions are expected after vaccination. Vaccination is the most effective way to prevent COVID-19. CDC and FDA will continue to monitor vaccine safety and will provide updates as needed to guide COVID-19 vaccination recommendations. 

VAERS is a national passive vaccine safety surveillance system, jointly managed by CDC and FDA, that monitors adverse events after vaccination (*7*). VAERS accepts reports from anyone, including health care providers,^§^ vaccine manufacturers, and members of the public. Symptoms, signs, and diagnostic findings in VAERS reports are assigned Medical Dictionary for Regulatory Activities (MedDRA) preferred terms by VAERS staff members.^¶^ VAERS reports are classified as serious if any of the following are reported: hospitalization, prolongation of hospitalization, life-threatening illness, permanent disability, congenital anomaly or birth defect, or death.** Reports of serious adverse events receive follow-up by VAERS staff members to obtain additional information, including medical records. For reports of death, death certificates and autopsy reports are obtained, if available. CDC physicians reviewed all available information for each decedent to form an impression about cause of death. Reports of myocarditis and pericarditis after receipt of COVID-19 vaccine were identified by a search for selected MedDRA preferred terms (*7*); CDC staff members attempted to collect information about clinical course and recovery related to myocarditis and pericarditis from patients and health care providers.

CDC established v-safe,^††^ a voluntary smartphone-based active safety surveillance system, specifically to monitor adverse events after COVID-19 vaccination. Parents and guardians can enroll children in v-safe after either the first or second vaccine dose. Text message reminders for online health surveys are sent to parents or guardians to complete for a child.^§§^ Health surveys sent in the first week after vaccination included questions about local injection site and systemic reactions (mild, moderate, or severe)^¶¶^ and health impacts (i.e., whether the child was unable to perform normal daily activities, missed school, or received care from a medical professional because of new symptoms or conditions). CDC’s v-safe call center contacted a parent or guardian when a report indicated that a child received medical care for new or worsening symptoms; completion of a VAERS report, if indicated, was encouraged.

VAERS and v-safe data collected during November 3–December 19, 2021 among children aged 5–11 years who received Pfizer-BioNTech COVID-19 vaccine were analyzed and described overall and by sex, age group, and race/ethnicity. Among 5,277 VAERS reports received for children aged 5–11 years who received Pfizer-BioNTech COVID-19 vaccine, 1,028 (19.5%) were excluded from this analysis because vaccination occurred before authorization for use in this age group or date of vaccination was unknown. SAS software (version 9.4; SAS Institute) was used to conduct all analyses. These activities were reviewed by CDC and conducted consistent with applicable federal law and CDC policy.***

## Review of VAERS Data

During November 3–December 19, 2021, VAERS received and processed 4,249 reports of adverse events (Table 1) for children aged 5–11 years who received Pfizer-BioNTech COVID-19 vaccine^†††^; the median age was 8 years, and 1,896 (44.6%) reports were for males. Most children (4,143; 97.5%) received Pfizer-BioNTech COVID-19 vaccine alone; seasonal influenza vaccine was the most frequently simultaneously administered vaccine (91 [2.1%] children).

**TABLE 1:** Adverse event reports among children aged 5–11 years who received Pfizer-BioNTech COVID-19 vaccine, by selected demographic characteristics and reported symptoms (N = 4,249) — Vaccine Adverse Event Reporting System, United States, November 3–December 19, 2021

Characteristic	Total, %	Nonserious, %	Serious, %*
	(N = 4,249)	(n = 4,149)	(n = 100)
**Sex**			
Female	45.0	45.1	39.0
Male	44.6	44.2	61.0
Unknown	10.4	10.7	0
**Age range, yrs (median)**	5–11 (8)	5–11 (8)	5–11 (9)
**Ethnicity**			
Hispanic or Latino	11.0	10.9	16.0
Non-Hispanic or Latino	40.0	39.7	56.0
Unknown ethnicity	48.9	49.4	28.0
**Race**			
American Indian or Alaska Native	0.6	0.6	0
Asian	4.0	4.0	7.0
Black	4.1	4.2	2.0
Native Hawaiian or Other Pacific Islander	0.2	0.2	0
White	39.5	39.2	52.0
Multiracial	2.2	2.1	9.0
Other	7.1	7.1	4.0
Unknown race	42.3	42.7	26.0

**Abbreviation**: VAERS = Vaccine Adverse Event Reporting System.

* VAERS reports are classified as serious if any of the following are reported: hospitalization or prolongation of hospitalization, life-threatening illness, permanent disability, congenital anomaly or birth defect, or death.

Overall, 4,149 (97.6%) VAERS reports were for nonserious events, and 100 (2.4%) were for serious events. The median age of children with reports of nonserious events was 8 years, and 1,835 (44.2%) of these reports were for males. The most commonly reported nonserious events were related to vaccine administration (some without any adverse event), including no adverse event (1,157; 27.9%), product preparation issue (925; 22.3%), and incorrect dose administered (675; 16.3%), (Table 2). The median age of children with reports of serious events was 9 years, and 61 (61.0%) reports were among males. The most commonly reported conditions and diagnostic findings among the 100 reports of serious events were fever (29; 29.0%), vomiting (21; 21.0%), and increased troponin^§§§^ (15; 15.0%). Among 12 serious reports of seizure, one child experienced syncope (not seizure) and another child potentially experienced syncope, two children experienced febrile seizure, one child had a history of seizures, two children had a potentially evolving seizure disorder, and five children experienced new-onset seizures. Among 15 preliminary reports of myocarditis identified during the analytic period, 11 were verified (by provider interview or medical record review) and met the case definition for myocarditis^¶¶¶^; of these 11 children, seven recovered, and four were recovering at time of the report. VAERS received two reports of death during the analytic period; both are under review. These deaths occurred in two females, aged 5 and 6 years, both of whom had complicated medical histories and were in fragile health before vaccination. None of the data suggested a causal association between death and vaccination.

**TABLE 2:** Most frequent symptoms, signs, diagnostic results, and conditions by MedDRA preferred term* reported to the Vaccine Adverse Event Reporting System among children aged 5–11 years after receipt of Pfizer-BioNTech COVID-19 vaccine (N = 4,249) — United States, November 3–December 19, 2021

Symptom, sign, diagnostic result, or condition (MedDRA PT)	No. reporting	% Reporting
**Nonserious reports (n = 4,149)**		
No adverse event^†^	1,157	27.9
Product preparation issue	925	22.3
Incorrect dose administered	675	16.3
Underdose	324	7.8
Vomiting	316	7.6
Fever	291	7.0
Headache	255	6.2
Syncope	255	6.2
Dizziness	244	5.9
Fatigue	201	4.8
Nausea	192	4.6
Urticaria	186	4.5
Rash	166	4.0
Pallor	151	3.6
Product storage error	146	3.5
**Serious reports** ^§^ **(n = 100)**		
Fever	29	29.0
Vomiting	21	21.0
Troponin increased	15	15.0
Chest pain	12	12.0
Echocardiogram normal	12	12.0
Blood test	11	11.0
C-reactive protein increased	11	11.0
SARS-CoV-2 test negative	11	11.0
Appendicitis	10	10.0
Electrocardiogram normal	10	10.0
Headache	10	10.0
Rash	10	10.0
Seizure	10	10.0
Intensive care	9	9.0
Full blood count normal	8	8.0

**Abbreviations:** MedDRA PT = Medical Dictionary for Regulatory Activities preferred term; VAERS = Vaccine Adverse Event Reporting System.

* Signs and symptoms in VAERS reports are assigned MedDRA PTs by VAERS staff members. Each VAERS report might be assigned more than one MedDRA PT, which can include normal diagnostic findings. A MedDRA PT does not indicate a medically confirmed diagnosis. Reports of myocarditis and seizure were identified using a combination of MedDRA PTs; in some cases, reports of myocarditis (identified by fulfilling criteria of the CDC working case definition of myocarditis) and seizure did not have the MedDRA PT “myocarditis” or “seizure” assigned to them. https://www.meddra.org/how-to-use/basics/hierarchy

^†^ Reports of no adverse event were accompanied by product preparation issue, incorrect dose administered, or underdose.

^§^ VAERS reports are classified as serious if any of the following are reported: hospitalization, prolongation of hospitalization, life-threatening illness, permanent disability, congenital anomaly or birth defect, or death; MedDRA PTs are included with serious reports when they occur in association with the criteria for serious classification (i.e., radiologic or laboratory tests that occur during a hospitalization).

## Review of v-safe Data

During November 3–December 19, 2021, v-safe enrolled 42,504 children aged 5–11 years who received Pfizer-BioNTech COVID-19 vaccine (Table 3); second dose information was available for 29,899 (70.3%) of these children. During the week after receipt of dose 1, local (23,290; 54.8%) and systemic (14,734; 34.7%) reactions were frequently reported; systemic reactions were more frequently reported during the week after dose 2 (12,223; 40.9%) than dose 1. Reactions were reported most frequently on the day after vaccination for both doses. The most frequently reported reactions after either dose were injection site pain, fatigue, and headache. Fever was more frequently reported after dose 2 (4,001; 13.4%) than dose 1 (3,350; 7.9%).

**TABLE 3:** Reactions reported for children aged 5–11 years (N = 42,504) who completed at least one v-safe health check-in survey on days 0–7 after receiving Pfizer-BioNTech COVID-19 vaccine — United States, November 3–December 19, 2021

Event	% of v-safe enrollees reporting reaction or health impact*	
	Dose 1 (N = 42,504)	Dose 2 (n = 29,899)
**Any injection site reaction**	54.8	57.5
Itching	3.8	3.7
Pain	52.7	55.8
Redness	3.7	4.4
Swelling	3.9	4.9
**Any systemic reaction**	34.7	40.9
Abdominal pain	5.1	6.4
Myalgia	7.1	10.2
Chills	3.9	6.8
Diarrhea	2.6	2.2
Fatigue	20.1	25.9
Fever	7.9	13.4
Headache	13.9	19.8
Joint pain	2.1	2.9
Nausea	5.0	6.9
Rash	1.2	1.0
Vomiting	2.3	2.7
**Any health impact**	10.9	15.1
Unable to perform normal daily activities	5.1	7.4
Unable to attend school	7.9	10.9
Needed medical care	1.2	1.1
Telehealth	0.3	0.2
Clinic	0.6	0.6
Emergency visit	0.1	0.1
Hospitalization	0.02	0.02

* Percentage of enrollees who reported a reaction or health impact at least once during days 0–7 post-vaccination.

Approximately 5.1% of parents reported that their child was unable to perform normal daily activities on the day after receipt of dose 1, and 7.4% after receipt of dose 2. Approximately 1% of parents reported seeking medical care in the week after vaccination; most medical care was received via a clinic appointment (441; 0.6%). Fourteen (0.02%) children reportedly received care at a hospital; information regarding reason for hospitalization was available for five children and included appendicitis (two), vomiting and dehydration (one), respiratory infection (one), and retropharyngeal cellulitis (one). Parents and guardians of all hospitalized children were contacted; two parents completed VAERS reports, and one revealed hospitalization was reported in error.

## Discussion

This report provides preliminary safety findings from VAERS and v-safe data collected during the administration of approximately 8 million doses of Pfizer-BioNTech COVID-19 vaccine to children aged 5–11 years. The findings summarized in this report are similar to the safety data from preauthorization trials for Pfizer-BioNTech COVID-19 vaccine administered to children aged 5–11 years (*4*,*5*). Trial participants who received Pfizer-BioNTech COVID-19 vaccine frequently reported local (86.2%) and systemic (66.6%) reactions that were mostly mild (i.e., did not interfere with normal daily activities) or moderate (some interference with normal daily activities); no serious adverse events judged to be related to vaccination were reported (*3*).

Among VAERS reports for children aged 5–11 years who received Pfizer-BioNTech COVID-19 vaccine, approximately 97% were nonserious. The most common adverse events reported to VAERS in the age group were related to administration error. This age group is the first to receive a smaller dosage of mRNA (10 *μ*g) than that recommended for persons aged ≥12 years (30 *μ*g), and administration errors are not unexpected. Most reports of administration errors often mentioned that no adverse event was associated with receipt of an incorrect dose.

Myocarditis is a rare and serious adverse event that has been associated with mRNA-based COVID-19 vaccines; reporting rates for vaccine-associated myocarditis appears highest among males aged 12–29 years (*8*). To date, myocarditis among children aged 5–11 years appears rare; 11 verified VAERS reports have been received after administration of approximately eight million vaccine doses, and, in an active vaccine safety surveillance system, no chart-confirmed reports of myocarditis were observed during the 1–21 days or 1–42 days after 333,000 vaccine doses were administered to children of the same age (*6*) These cases appear consistent with other reports of myocarditis after mRNA COVID-19 vaccination regarding time to symptom onset and a mild clinical course (*9*). Two deaths after Pfizer-BioNTech COVID-19 vaccine were reported for children with multiple chronic medical conditions; on initial review, no data were found that would suggest a causal association between death and vaccination.

Local (57.5%) and systemic (40.9%) reactions after receipt of dose 2 of Pfizer-BioNTech COVID-19 vaccination among v-safe registrants aged 5–11 years were less frequently reported than reactions reported among children and adolescents aged 12–15 years (local 62.4%; systemic, 63.4%) (*9*). Fourteen v-safe registrants aged 5–11 years were reported to have been hospitalized after vaccination. V-safe does not directly record diagnoses associated with hospitalization; however, parents and guardians can include supplemental text for each health check-in. Whether hospitalization was the result of vaccination could not be determined; however, all parents and guardians who reported a child’s hospitalization were contacted and encouraged to complete a VAERS report. Two parents completed a VAERS report on behalf of a child who was reported to v-safe to have been hospitalized.

The findings in this report are subject to at least four limitations. First, VAERS is a passive surveillance reporting system and is subject to reporting biases and underreporting, especially of nonserious events (*8*). Second, data on race/ethnicity were not provided in >40% of VAERS reports. Third, v-safe is a voluntary program; as a result, v-safe data might not be representative of the vaccinated population. Finally, these data are limited by the short surveillance period and might change as safety monitoring continues and more doses are administered to children aged 5–11 years.

Vaccination is the most effective way to prevent COVID-19 infection. ACIP recommends the Pfizer-BioNTech COVID-19 vaccine for children aged 5–11 years for the prevention of COVID-19 (*10*). Preliminary safety findings are similar to those described in the clinical trials. Parents and guardians of children aged 5–11 years vaccinated with Pfizer-BioNTech COVID-19 vaccine should be advised that local and systemic reactions are expected after vaccination. CDC and FDA will continue to monitor vaccine safety and will provide updates as needed to guide COVID-19 vaccination recommendations.

**Summary**

**What is already known about this topic?**

In preauthorization trials for Pfizer-BioNTech (BNT162b2) COVID-19 vaccine, vaccinated children aged 5–11 years reported mild to moderately severe local and systemic reactions; no serious vaccination-related events were noted.

**What is added by this report?**

After authorization of Pfizer-BioNTech COVID-19 vaccine for children aged 5–11 years during October 2021, and administration of approximately 8 million doses, local and systemic reactions after vaccination were commonly reported to VAERS and v-safe for vaccinated children aged 5–11 years. Serious adverse events were rarely reported.

**What are the implications for public health practice??**

Parents and guardians of children aged 5–11 years should be advised that local and systemic reactions are expected after vaccination with Pfizer-BioNTech COVID-19 vaccine and are more common after the second dose.

## Footnotes

*
https://www.cdc.gov/coronavirus/2019-ncov/vaccines/safety/vsafe.html

†
https://covid.cdc.gov/covid-data-tracker/#vaccination-demographic (Accessed December 19, 2021).

§ Under COVID-19 vaccine EUA requirements, health care providers are required to report certain adverse events after vaccination to VAERS, including death. https://www.cdc.gov/vaccinesafety/ensuringsafety/monitoring/vaers/index.html

¶ Each VAERS report might be assigned more than one MedDRA preferred term. A MedDRA-coded event does not indicate a medically confirmed diagnosis. https://www.meddra.org/how-to-use/basics/hierarchy

** Based on the Code of Federal Regulations Title 21. https://www.accessdata.fda.gov/scripts/cdrh/cfdocs/cfcfr/cfrsearch.cfm?fr=312.32

††
https://vsafe.cdc.gov

§§ Children and adolescents aged ≤15 years must be enrolled by a parent or guardian and cannot self-enroll. Children aged 5–11 years might be enrolled in v-safe if they were vaccinated on or after November 3, 2021. Health check-ins are sent via text messages that link to web-based surveys on days 0–7 after vaccination; then weekly through 6 weeks after vaccination; and then 3, 6, and 12 months after vaccination.

¶¶ Parents and guardians who participate in v-safe use the following definitions to describe the severity of a child’s symptoms: mild (noticeable, but not problematic), moderate (limit normal daily activities), or severe (make daily activities difficult or impossible).

*** 45 C.F.R. part 46, 21 C.F.R. part 56; 42 U.S.C. Sect. 241(d); 5 U.S.C. Sect. 552a; 44 U.S.C. Sect. 3501 et seq.

††† Processed VAERS reports are those that have been coded using MedDRA, have been deduplicated, and have undergone standard quality assurance and quality control review.

§§§ Troponins are proteins found in myocytes (heart muscle cells). Troponin levels typically are measured as part of the evaluation of chest pain or other symptoms of possible myocardial damage.

¶¶¶ Acute myocarditis was defined as presence of signs and symptoms (one or more new or worsening of the following: chest pain/pressure/discomfort, dyspnea/shortness of breath/pain with breathing, palpitations, or syncope; or two or more of the following in children aged ≤11 years: irritability, vomiting, poor feeding, tachypnea, or lethargy); and one or more new finding of elevated troponin, electrocardiogram findings consistent with myocarditis, abnormal cardiac function or wall motion on echocardiogram, cardiac magnetic resonance imaging findings consistent with myocarditis, or histopathologic findings consistent with myocarditis; and no other identifiable cause for these findings.
